# Decrease in Temporal Gyrus Gray Matter Volume in First-Episode, Early Onset Schizophrenia: An MRI Study

**DOI:** 10.1371/journal.pone.0040247

**Published:** 2012-07-03

**Authors:** Jinsong Tang, Yanhui Liao, Bing Zhou, Changliang Tan, Weiqing Liu, Dong Wang, Tieqiao Liu, Wei Hao, Liwen Tan, Xiaogang Chen

**Affiliations:** 1 Institute of Mental Health, the Second Xiangya Hospital, Central South University, Changsha, Hunan, People’s Republic of China; 2 Department of Radiology, the Second Xiangya Hospital, Central South University, Changsha, Hunan, People’s Republic of China; University of Missouri-Kansas City, United States of America

## Abstract

**Background:**

Loss of gray matter has been previously found in early-onset schizophrenic patients. However, there are no consistent findings between studies due to different methods used to measure grey matter volume/density and influences of confounding factors.

**Methods:**

The volume of gray matter (GM) was measured in 29 first episode early-onset schizophrenia (EOS) and 34 well-matched healthy controls by using voxel-based morphometry (VBM). Psychotic symptoms were assessed using the Positive and Negative Syndrome Scale (PANSS). The correlations between the GM volume and PANSS scores, age of psychosis onset, duration of psychosis, and chlorpromazine (CPZ) equivalent value were investigated.

**Results:**

Relative to healthy subjects, the patients with first episode EOS showed significantly lower GM volume in the left middle and superior temporal gyrus. The loss of GM volume negatively correlated with PANSS-positive symptoms (p = 0.002), but not with PANSS-negative symptoms, PANSS-general psychopathology, and PANSS-total score. No significant correlation was found between GM volume and age of psychosis onset, duration of psychosis, and CPZ equivalent value.

**Conclusion:**

Patients with first episode EOS have evidence of reduced GM in the left middle and superior temporal gyrus. Structural abnormalities in the left middle and superior temporal gyrus may contribute to the pathophysiology of schizophrenia.

## Introduction

Schizophrenia is a severe psychiatric illness found in about 1% of the population. This chronic disorder is characterized by pathological manifestations such as hallucinations, disorganized thinking, loss of goal-directed behaviors, social withdrawal, and cognitive deficits [1]. Schizophrenia is generally accepted as a neurodevelopmental disorder. Schizophrenia generally begins early on in the life of the affected and tends to persist throughout life with symptoms sometimes worsening with time [2]. Therefore, studies on early-onset schizophrenia offer a unique opportunity to explore the etiology of this major mental disorder.

Early-onset schizophrenia (EOS) is defined as the first-episode of schizophrenia occurring when the subject is younger than 18 years of age. Thus, studies of EOS can benefit from minimal confounding factors such as antipsychotic treatment, shorter duration of psychosis, and narrow age range. There is evidence that antipsychotic medications can affect brain structure [3]. However, several studies reported no significant correlation between FA and dosage of antipsychotic medication [4], [5]. Therefore, whether the use of medication is a major confounding factor in schizophrenia research remains controversial. Several studies showed an inverse relationship between duration of untreated psychosis and gray matter loss in patients with schizophrenia [6], [7]. However, a few studies that looked at volumetric measurements have found no association between duration of untreated psychosis and gray matter changes in schizophrenia [8], [9]. Age is another potential confounding factor. Age may have a differential effect on brain structure in schizophrenia [10]. For example, it was reported that age-related changes in gray matter are dynamic throughout normal adolescence [11]–[13]. Even so, antipsychotic treatment, duration of psychosis, and age of patients are usually considered to be confounders when researching the structure and function of the brain in schizophrenia. Thus, the examination of structural brain changes in minimally treated subjects, such as first-episode EOS, will minimize the effects of these confounding factors.

Neuroimaging studies clearly indicate that early-onset schizophrenia is associated with neuroanatomical abnormalities. Several recent MRI studies of early-onset schizophrenia have identified brain abnormalities in the parietal cortex [14] and cingulate cortex [15] in white matter, and the superior parietal cortices of gray matter [16], [17] with changes progressing more towards the prefrontal and temporal cortices by adolescence [18]. In addition, Arango et al. study on children and adolescents with first-episode psychosis showed that the primary initial changes in brain structure were in the frontal lobe and left parietal cortex in gray matter over time [19]. In EOS, the rate of grey matter loss appears higher than in other forms of schizophrenia with large changes found in parietal brain regions, extending anteriorly into temporal lobes [17] and superior medial frontal grey matter loss later reaching the cingulate gyrus [20]. Although there are some consistencies in findings [21], an obvious divergence in findings between studies exists. The inconsistency in neuroanatomical studies of schizophrenia may be related to differences in the methods of measuring grey matter volume/density, such as whole brain versus region of interest (ROI), and differences in analysis, such as ROI versus voxel-based morphometry (VBM), as well as the influences of confounding factors. Thus, the accumulation of data on neuroanatomical abnormalities in EOS patients is valuable and may help identify the etiology of schizophrenia.

Cognitive deficits are the core characteristics of schizophrenia. Temporal lobe regions of gray matter are associated with auditory hallucinations, thought disorder, and memory dysfunction. In this study,psychotic symptoms were assessed using the Positive and Negative Syndrome Scale (PANSS). The volume of gray matter (GM) was measured by using voxel-based morphometry (VBM). The correlations between the GM volume and PANSS scores, age of psychosis onset, duration of psychosis, and chlorpromazine (CPZ) equivalent value were investigated.

## Methods

### 1. Ethics Statement

The protocols used in this study was approved by the university ethics committee (The Second Xiangya Hospital of Central South University Review Board,No. S078, 2006) and the studies were carried out in accordance with the Declaration of Helsinki. Subjects were fully informed about the measurement and MRI scanning in the study. Written informed consent forms were obtained from all subjects and their parents.

### 2. Subjects

Twenty-nine first-episode, early-onset schizophrenic patients (13 females, 16 males) with an age of 16.5±0.9 years and average education of 9.7±1.4 years were recruited at the Institute of Mental Health of the Second Xiangya Hospital, Central South University. All participants met the following inclusion criteria: 1) met the DSM-IV-TR criteria for schizophrenia (Diagnostic and Statistical Manual of Mental Disorders, fourth edition, text revision, American psychiatric association, 2000); 2) aged 12 to 19 years old; 3) onset of schizophrenia before the 18^th^ birthday; 4) no comorbid Axis I diagnosis; and 5) no mental retardation. Schizophrenia was diagnosed by clinical psychiatrists using the Structured Clinical Interview for DSM-IV-TR, patient version (SCID-I/P). These patients were interviewed again six months later to determine a final diagnosis of schizophrenia. All patients were recruited during an acute psychotic episode. Patients with concurrent psychiatric disorders and history of major neurological or physical disorders that could lead to an altered mental state were excluded. Thirty-four healthy subjects with matched age (16.6±0.8 years), gender (16 females, 18 males), and average education (9.7 years) were recruited by advertisement as a control group. Control subjects were identified: 1) without any known psychiatric condition; 2) without a history of major physical or neurological illness or substance abuse; 3) without a family history of psychosis in their first-degree relatives. All patients and control subjects were right-handed.

### 3. Imaging Acquisition

Magnetic resonance imaging was performed on a 1.5T GE MRI scanner (Twin-speed, Milwaukee) at the Second Xiangya Hospital, Central South University. A standard birdcage head coil was used, along with foam pads for limiting head motion and reducing scanner noise. High-resolution whole brain volume T1-weighted images were acquired sagittally with a 3D spoiled gradient echo (SPGR) pulse sequence (repetition time  = 12.1 ms; echo time  = 4.2 ms; flip angle  = 15; field of view  = 240 mm×240 mm; acquisition matrix  = 256×256; slice thickness  = 1.8 mm; gap  = 0 mm; number of excitations  = 2; 172 slices).

### 4. MRI Data Analysis

All structural data were processed with voxel-based morphometry toolbox (VBM5.1) (http://dbm.neuro.uni-jena.de/vbm) with the Statistical Parametric Mapping (SPM) 5 software package (http://www.fil.ion.ucl.ac.uk/spm ). The VBM toolbox combines tissue segmentation, bias correction, and spatial normalization into a unified model [22]. A Hidden Markov Random Field (HMRF) model was used to introduce spatial constraints into the segmentation process to improve accuracy of tissue segmentation [23]. Images were multiplied (modulated) by the Jacobian determinants from the normalization step to preserve volume information. Modulated gray matter images were smoothed with an 8 mm FWHM Gaussian kernel for statistical analyses.

To examine differences in regional gray matter volume between groups, a voxel-wise two-sample t-test analysis was carried out in SPM5. The total gray matter volume, age, gender, and years of education were treated as confounding covariates. An absolute threshold mask of 0.1 was used to avoid possible edge effects around the border between gray matter and white matter. Clusters of 100 voxels (a cluster size equal to 1×1×1 mm^3^) or greater, surviving a false discovery rate (FDR) corrected threshold of *p*<0.05 were considered significant.

The multiple regression analysis was performed as previously described [5]. Briefly, a multiple regression was carried out in SPM5 on the patients that had chlorpromazine equivalents (n = 23) to test the relationship between medication and regional gay matter volume while eliminating the effects of age, gender, years of education, and total gray matter volume An absolute threshold mask of 0.1 was used to avoid possible edge effects around the border between gray matter and white matter. Clusters of 100 voxels (a cluster size equal to 1×1×1 mm^3^) or greater, surviving a false discovery rate (FDR) corrected threshold of p<0.05 were considered significant.

### 5. Positive and Negative Syndrome Scale (PANSS)

Patients with positive diagnosis of schizophrenia were subjected to the PANSS interview and were then assessed on the PANSS scales [24]. The assessments were conducted by clinical psychiatrists with intensive training in the PANSS interview and rating methods.

### 6. Statistical Analyses

Statistical analyses of demographic data were conducted with SPSS 15.0 software (SPSS, Chicago, Illinois). Independent-sample t test and χ2 test were used to compare demographic data between two groups. In addition, we extracted the mean volumes of the clusters that had shown differences between groups in VBM analysis. Mean volume measurements were calculated using MarsBar 0.41 (http://marsbar.sourceforge.net/) and log_roi_batch v2.0 (http://www.aimfeld.ch). Correlations between mean volumes of the clusters and clinical factors including PANSS scores, age of psychosis onset, duration of psychosis, and chlorpromazine (CPZ) equivalent value were calculated by partial correlation analysis controlling for total gray matter volume, age and gender (*p<*0.05).

## Results

### 1. Demographic Findings

Demographic and clinical data for the two groups are shown in Table 1. We included 29 patients with first-episode EOS and 34 healthy controls well-matched in gender, levels of education, ethnicity, and handedness. All patients were diagnosed with schizophrenia according to DSM-IV-TR. All recruited patients had a short duration of illness (9.3±4.6 months) at the entry of the study. Except for six patients who did not receive medications, 23 patients received atypical antipsychotic medications and the mean dosage in chlorpromazine equivalent was 252.2 mg/day (S.D. 189.2 mg/day; range: 0–660 mg/day) at the time of scanning (risperidone [n = 13], clozapine [n = 1], sulpiride [n = 5], or quetiapine [n = 4]). Patients and control subjects were statistically similar in terms of gender composition, age, handedness, and educational level (Table 1).

**Table 1:** Demographic and clinical characteristics of EOS patients and control subjects.

	EOS patients	Control subjects	P value
Demographic variables			
Cases	29	34	
Years of Age, mean±SD	16.5±0.9	16.6±0.8	0.69^a^
Sex, Male/Female	13/16	16/18	0.86^b^
Subjects’ education, years, mean±SD	9.7±1.4	9.7±0.7	0.78^a^
Han Chinese	29	34	
Left/Right-handed	0/29	0/34	
clinical variablies			
Onset age (year), mean±SD	15.7±1.0	―	
Course of disease (month)	9.3±4.6	―	
PANSS-positive symptoms	22.3±2.8	―	
PANSS-negative symptoms	20.8±3.0	―	
PANSS-general psychopathology	34.5±3.7	―	
PANSS-total score	77.7±5.7	―	
CPZ-eqquivalent	252.2±189.2	―	

a T-test.

b Chi-square tests.

EOS: Early-onset schizophrenia.

CPZ Equivalent: average chlorpromazine equivalent dosage for those taking antipsychotic medications.

### 2. Group Differences in Regional Gray Matter Volume

**Figure 1:**
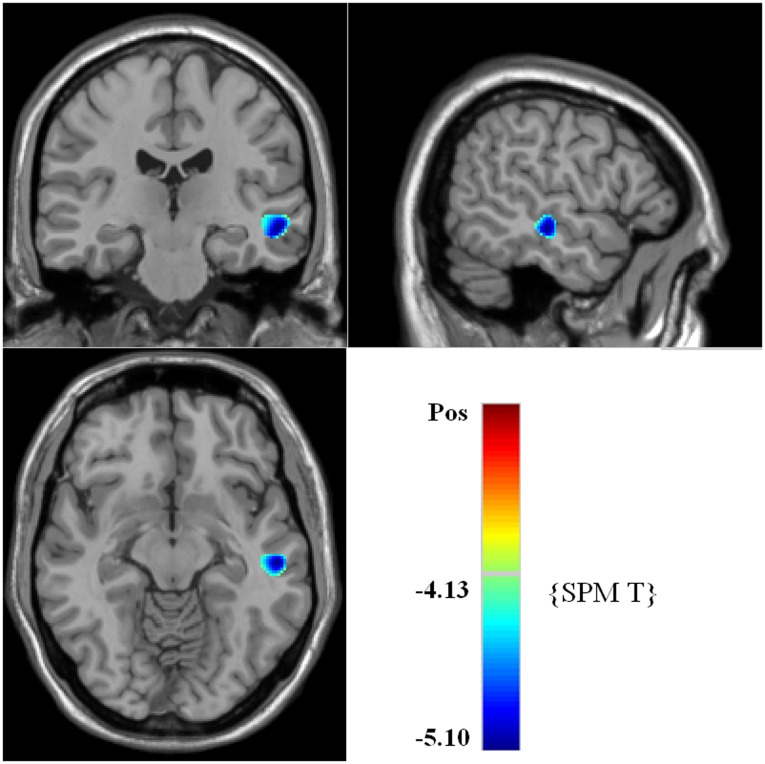
Brian maps of representative axial slices showing the differences of gray matter volume between patients with EOS group and control group^a,b^. ^a^ Voxels of reduced gray matter volume in left middle and superior temporal gyrus (with blue colour) in 29 First-Episode early-onset Schizophrenia (EOS) patients revealed by SPM5 analysis. Numbers indicate Montreal Neurological Institute *z*-coordinates. ^b^ Cluster size is in voxels. All clusters shown exceed a FDR corrected statistical threshold of cluster-level P<0.05 and a cluster size threshold of 100 consecutive voxels.

VBM analysis of gray matter volume showed significant reduction in the volume of left middle and superior temporal gyrus in patients with first-episode EOS compared to healthy subjects (Fig. 1A, Table 2). In contrast, no region showed significant increase in gray matter volume in patients.

**Table 2:** Regions of reduced gray matter volumes of left middle and superior temporal gyrus in EOS group in comparison with the control group.

Brodmann area	Anatomical Region	Cluster Size (No. Voxel)	Voxel level P		MNI (mm)			Peak Z- value
			FWE corrected	FDR corrected	X	Y	Z	
21	Left Middle and Superior Temporal Gyrus	353	0.020	0.018	−54	−22	−11	4.61

FDR: false discovery rate; FWE: family-wise error; MNI: montreal neurological institute. FDR corrected p<0.05, cluster size = 100. First-Episode early-onset schizophrenia (EOS) patients, *n* = 29; healthy controls, *n* = 34.

### 3 Medication Effects on Gray Matter

No regions in the gray matter with increases or decreases in volume correlated with chlorpromazine equivalents in multiple regression analysis.

### 4. Relationship between Clinical Factors and Volumes of Left Middle and Superior Temporal Gyrus

Partial correlation analyses were used to examine the correlations between gray matter volume of left middle and superior temporal gyrus and PANSS-positive symptoms, PANSS-negative symptoms, PANSS-general psychopathology, PANSS-total score, age of psychosis onset, duration of psychosis, CPZ equivalent in patients with EOS. The PANSS-positive symptoms negatively correlated with gray matter volume in left middle and superior temporal gyrus (partial correlation coefficient = −0.607, p = 0.002). We did not find any correlations between PANSS-negative symptoms, PANSS-general psychopathology, PANSS-total score, age of psychosis onset, duration of psychosis, CPZ equivalent, and gray matter volume of left middle and superior temporal gyrus.

## Discussion

Temporal lobe regions are associated with auditory hallucinations, thought disorder, and memory dysfunction, which are the key characteristics of schizophrenia [1]. In this study, a significant decrease in GM volume was observed in left middle and superior temporal gyrus of schizophrenic patients compared to healthy controls. Importantly, the volume of left middle and superior temporal gyrus negatively correlated with PANSS-positive symptoms. Our study highlighted the crucial role of temporal gyrus in the pathology of EOS.

Structural and functional abnormalities on gray and white matter were widely observed in patients with schizophrenia. Neuroanatomical abnormalities in other brain regions were also reported. Although there are some consistencies in findings to be observed [21], there is no highly consistent finding between studies. Relatively, reduction in parietal cortex was well established in both structural neuroimaging and functional studies of schizophrenia [5], [14], [17]. Particularly, using various VBM analyses, significant decreases in the volume of left medial and left middle frontal gyrus were observed in first-episode EOS [25]. In contrast, Pagsberg et al. study did not find GM volume deficits in first-episode EOS compared to healthy controls [26]. The results of the present study were established in a relatively large sample of first-episode EOS patients by VBM analysis. Significant decrease in gray matter volume in the left middle and superior temporal gyrus was observed in first-episode EOS patients compared to well-matched healthy controls. As mentioned above, changes in the parietal cortex are the main findings in early onset studies. Our findings may reflect the differences in the average onset age [17], methods of measuring grey matter volume/density [19], sample size [25] and the population. For example, most studies were conducted with a small sample size. Although VBM analysis has the advantage of searching for putative regions within the entire brain devoid of any bias, using VBM in a small sample to examine subtle neurobiological effects could result in insufficient statistical power to detect differences [25]. Other confounders, such as antipsychotic treatment, duration of psychosis, and age of patients also affect the results of brain structural and functional studies of schizophrenia.

The Positive and Negative Syndrome Scale (PANSS) is currently the most well-established scale in patients with schizophrenia for typological and dimensional assessments. The positive symptoms reflect a hyperdopaminergic state while the negative symptoms are thought to be the consequences of structural brain-deficit [27]. Previous studies demonstrated that PANSS scores negatively correlated with decreased FA in the corpus callosum of chronic schizophrenic patients [28] and decreased FA in right anterior cingulum of early-onset schizophrenic patients [29]. In this study, VBM analysis showed that the loss of grey matter volume in left middle and superior temporal gyrus negatively correlated with PANSS-positive symptoms, but not with PANSS-negative symptoms, PANSS-general psychopathology, and PANSS-total score. Therefore, the current finding might imply a loss of dopaminergic neuron or the reduction of their plasticity.

Interestingly, in this study, the decreased volume of left middle and superior temporal gyrus in first episode EOS showed no correlations with age of psychosis onset, duration of psychosis, or CPZ equivalent. Previous studies showed an association between duration of psychosis and gray matter loss in schizophrenic patients. Keshavan et al. study reported an inverse relationship between duration of untreated psychosis and left superior temporal gyrus volume [6]. However, a study of patients with first-episode EOS and short duration of illness can avoid possible confounding effects of chronic illness. In this study, the duration of illness was only 9.3±4.6 months. In addition, there is evidence that antipsychotic medication can affect brain structure [6], [7] and thereby act as a possible confounder in the search for the neuropathology of psychosis itself. The present study included a cohort of minimally treated first episode psychotic patients. No correlation between GM loss and CPZ equivalent was found. This suggested that the observed GM loss reflects the structural abnormalities of EOS itself. Notably, the gray matter loss in EOS appears dynamically with age. Loss of gray matter was found early in parietal brain regions extending anteriorly into temporal lobes [17] and with superior medial frontal gray matter loss later reaching the cingulate gyrus [20]. Importantly, abnormalities in temporal lobe structures, including the superior temporal gyrus and amygdala-hippocampal complex, play a crucial role in dysfunction of auditory functions, language processing, and in memory of patients with schizophrenia. Interestingly, our study revealed no correlation between age of psychosis onset and GM loss. This might be because there is a narrow age range (16.5±0.9 years) in the patients and healthy controls were well-matched in gender, levels of education, ethnicity, and handedness.

The accurate mechanism for GM loss is currently unclear. Several neurodevelopmental mechanisms have been used to explain the neuropathology of GM loss. One is the hypothesis of pathological extended pruning. This hypothesis is based on the observations that normal adolescent brain development is characterized by reduction of GM and expansion of WM, sculpting the brain into maturity [12], [17]. The typical onset of schizophrenia occurs during adolescence, which could be a consequence of a defect in this normal maturational process [30]. This defect may cause abnormal connectivity in brain networks that, in turn, lead to illness characterized by hallucinations, delusions, alogia, or decreased emotional expression. However, in this study, decrease in GM volume did not correlate with the age of psychosis onset and duration of psychosis, which does not seem to support the hypothesis. Another hypothesis suggests that GM loss is a consequence of a confounder, such as neuroleptic treatment. Studies of neuroleptic-treated animals have demonstrated that neuroleptic-treated animals have brain volume reductions [31], [32]. We have examined treatment effects in the current sample and no correlation between GM loss and CPZ equivalent was found, which does not agree with the hypothesis. Another possible hypothesis is the neuroplasticity mechanism. GM decreases that occur after the onset of schizophrenia is caused by diminished neuroplasticity and an impairment in activity-dependent neuroplasticity. Neuroplasticity affects spines and synapses, leading to shrinkage of neuropil without gliosis or neuronal loss [33], [34]. This hypothesis emphasizes the interaction between circumstance and genes responsible for the regulation of neurodevelopment and neuroplasticity. Here, circumstance might be a main driving force. It was hypothesized that environmental factors can regulate gene expression through an epigenetic mechanism that ultimately causes schizophrenia [35], [36]. In this study, the loss of GM volume negatively correlated with PANSS-positive symptoms, which may suggest a reduction in dopaminergic neuron plasticity.

In conclusion, loss of gray matter volume in left middle and superior temporal gyrus is a brain structural abnormality of first episode early onset schizophrenia and is negatively correlated with PANSS-positive symptoms. This loss of gray matter is not associated with age of psychosis onset, duration of psychosis, or CPZ equivalent value, suggesting that GM loss observed in this study represents the structural abnormalities of EOS itself. Our study highlighted the crucial role of middle temporal and superior gyrus in the pathology of EOS and the importance of the study with first episode early-onset schizophrenia and well-matched healthy controls.

## References

[1] Schultz SK, Andreasen NC (1999). Schizophrenia.. Lancet.

[2] [TIGHEST]Jung WH, Jang JH, Byun MS, An SK, Kwon JS (2010). Structural brain alterations inindividuals at ultra-high risk for psychosis: a review of magnetic resonance imaging studies and future directions.. J Korean Med Sci.

[3] Bangalore SS, Goradia DD, Nutche J, Diwadkar VA, Prasad KM (2009). Untreated illness duration correlates with gray matter loss in first-episode psychoses.. Neuroreport.

[4] Peters BD, Blaas J, de Haan L (2010). Diffusion tensor imaging in the early phase of schizophrenia: what have we learned?. J Psychiatr Res.

[5] Honea RA, Meyer-Lindenberg A, Hobbs KB, Pezawas L, Mattay VS (2008). Is gray matter volume an intermediate phenotype for schizophrenia? A voxel-based morphometry study of patients with schizophrenia and their healthy siblings.. Biol Psychiatry.

[6] Keshavan MS, Haas GL, Kahn CE, Aguilar E, Dick EL (1998). Superior temporal gyrus and the course of early schizophrenia: progressive, static, or reversible?. J Psychiatr Res.

[7] Velakoulis D, Wood SJ, Smith DJ, Soulsby B, Brewer W (2002). Increased duration of illness is associated with reduced volume in right medial temporal/anterior cingulate grey matter in patients with chronic schizophrenia.. Schizophr Res.

[8] Hoff AL, Sakuma M, Razi K, Heydebrand G, Csernansky JG (2000). Lack of association between duration of untreated illness and severity of cognitive and structural brain deficits at the first episode of schizophrenia.. Am J Psychiatry.

[9] Ho BC, Alicata D, Ward J, Moser DJ, O’Leary DS (2003). Untreated initial psychosis: relation to cognitive deficits and brain morphology in first-episode schizophrenia.. Am J Psychiatry.

[10] Bartzokis G, Beckson M, Lu PH, Nuechterlein KH, Edwards, et al (2001). Age-related changes in frontal and temporal lobe volumes in men: a magnetic resonance imaging study.. Arch Gen Psychiatry.

[11] Rapoport JL, Giedd JN, Blumenthal J, Hamburger S, Jeffries N (1999). Progressive cortical change during adolescence in childhood-onset schizophrenia. A longitudinal magnetic resonance imaging study.. Arch Gen Psychiatry.

[12] Sowell ER, Delis D, Stiles J, Jernigan TL (2001). Improved memory functioning and frontal lobe maturation between childhood and adolescence: a structural MRI study.. J Int Neuropsychol Soc.

[13] Gogtay N, Giedd JN, Lusk L, Hayashi KM, Greenstein D (2004). Dynamic mapping of human cortical development during childhood through early adulthood.. Proc Natl Acad Sci U S A.

[14] Kyriakopoulos M, Vyas NS, Barker GJ, Chitnis XA, Frangou S (2008). A diffusion tensor imaging study of white matter in early-onset schizophrenia.. Biol Psychiatry.

[15] Tang J, Liao Y, Zhou B, Tan C, Liu T (2010). Abnormal Anterior Cingulum Integrity in First Episode, Early Onset Schizophrenia: A Diffusion Tensor Imaging Study.. Brain Research.

[16] Kumra S, Robinson P, Tambyraja R, Jensen D, Schimunek C (2012). Parietal lobe volume deficits in adolescents with schizophrenia and adolescents with cannabis use disorders.. J Am Acad Child Adolesc Psychiatry.

[17] Thompson PM, Vidal C, Giedd JN, Gochman P, Blumenthal J (2001). Mapping adolescent brain change reveals dynamic wave of accelerated gray matter loss in very early-onset schizophrenia.. Proc Natl Acad Sci U S A.

[18] Gogtay N, Greenstein D, Lenane M, Clasen L, Sharp W (2007). Cortical brain development in nonpsychotic siblings of patients with childhood-onset schizophrenia.. Arch Gen Psychiatry.

[19] Arango C, Rapado-Castro M, Reig S, Castro-Fornieles J, González-Pinto A (2012). Progressive brain changes in children and adolescents with first-episode psychosis.. Arch Gen Psychiatry.

[20] Vidal CN, Rapoport JL, Hayashi KM, Geaga JA, Sui Y (2006). Dynamically spreading frontal and cingulate deficits mapped in adolescents with schizophrenia.. Arch Gen Psychiatry.

[21] Honea R, Crow TJ, Passingham D, Mackay CE (2005). Regional deficits in brain volume in schizophrenia: a meta-analysis of voxel-based morphometry studies.. Am J Psychiatry.

[22] Ashburner J, Friston KJ (2005). Unified segmentation.. Neuroimage.

[23] Cuadra MB, Cammoun L, Butz T, Cuisenaire O, Thiran JP (2005). Comparison and validation of tissue modelization and statistical classification methods in T1-weighted MR brain images.. IEEE Trans Med Imaging.

[24] Kay SR, Fiszbein A, Opler LA (1987). The positive and negative syndrome scale (PANSS) for schizophrenia.. Schizophrenia Bulletin.

[25] Janssen J, Reig S, Parellada M, Moreno D, Graell M (2008). Regional gray matter volume deficits in adolescents with first-episode psychosis.. J Am Acad Child Adolesc Psychiatry.

[26] Pagsberg AK, Baaré WF, Raabjerg Christensen AM, Fagerlund B (2007). Structural brain abnormalities in early onset first-episode psychosis.. J Neural Transm.

[27] Emsley R, Rabinowitz J, Torreman M (2003). RIS-INT-35 Early Psychosis Global Working Group. The factor structure for the Positive and Negative Syndrome Scale (PANSS) in recent-onset psychosis.. Schizophr Res.

[28] Kong X, Ouyang X, Tao H, Liu H, Li L (2011). Complementary diffusion tensor imaging study of the corpus callosum in patients with first-episode and chronic schizophrenia.. J Psychiatry Neurosci.

[29] Skelly LR, Calhoun V, Meda SA, Kim J, Mathalon DH (2008). Diffusion tensor imaging in schizophrenia: relationship to symptoms.. Schizophr Res.

[30] Andreasen NC, Nopoulos P, Magnotta V, Pierson R, Ziebell S (2011). Progressive brain change in schizophrenia: a prospective longitudinal study of first-episode schizophrenia.. Biol Psychiatry.

[31] Konopaske GT, Dorph-Petersen KA, Pierri JN, Wu Q, Sampson AR (2007). Effect of chronic exposure to antipsychotic medication on cell numbers in the parietal cortex of macaque monkeys.. Neuropsychopharmacology.

[32] Konopaske GT, Dorph-Petersen KA, Sweet RA, Pierri JN, Zhang W (2008). Effect of chronic antipsychotic exposure on astrocyte and oligodendrocyte numbers in macaque monkeys.. Biol Psychiatry.

[33] Agartz I, Sedvall GC, Terenius L, Kulle B, Frigessi A (2006). BDNF gene variants and brain morphology in schizophrenia.. Am J Med Genet B Neuropsychiatr Genet.

[34] Szeszko PR, Lipsky R, Mentschel C, Robinson D, Gunduz-Bruce H (2005). Brain-derived neurotrophic factor val66met polymorphism and volume of the hippocampal formation.. Mol.

[35] Tsuang MT, Bar JL, Stone WS, Faraone SV (2004). Gene-environment interactions in mental disorders.. World Psychiatry.

[36] Schumacher A, Petronis A (2006). Epigenetics of complex diseases: from general theory to laboratory experiments.. Curr Top Microbiol Immunol.

